# Corporate Philanthropy, Political Influence, and Health Policy

**DOI:** 10.1371/journal.pone.0080864

**Published:** 2013-11-27

**Authors:** Gary J. Fooks, Anna B. Gilmore

**Affiliations:** Department for Health, University of Bath, Bath, United Kingdom; The National Institute for Health Innovation, New Zealand

## Abstract

**Background:**

The Framework Convention of Tobacco Control (FCTC) provides a basis for nation states to limit the political effects of tobacco industry philanthropy, yet progress in this area is limited. This paper aims to integrate the findings of previous studies on tobacco industry philanthropy with a new analysis of British American Tobacco's (BAT) record of charitable giving to develop a general model of corporate political philanthropy that can be used to facilitate implementation of the FCTC.

**Method:**

Analysis of previously confidential industry documents, BAT social and stakeholder dialogue reports, and existing tobacco industry document studies on philanthropy.

**Results:**

The analysis identified six broad ways in which tobacco companies have used philanthropy politically: developing constituencies to build support for policy positions and generate third party advocacy; weakening opposing political constituencies; facilitating access and building relationships with policymakers; creating direct leverage with policymakers by providing financial subsidies to specific projects; enhancing the donor's status as a source of credible information; and shaping the tobacco control agenda by shifting thinking on the importance of regulating the market environment for tobacco and the relative risks of smoking for population health. Contemporary BAT social and stakeholder reports contain numerous examples of charitable donations that are likely to be designed to shape the tobacco control agenda, secure access and build constituencies.

**Conclusions and Recommendations:**

Tobacco companies' political use of charitable donations underlines the need for tobacco industry philanthropy to be restricted via full implementation of Articles 5.3 and 13 of the FCTC. The model of tobacco industry philanthropy developed in this study can be used by public health advocates to press for implementation of the FCTC and provides a basis for analysing the political effects of charitable giving in other industry sectors which have an impact on public health such as alcohol and food.

## Introduction

The once confidential nature of industry documents makes them a particularly valuable source of data for analysing the thinking behind company policies on charitable contributions.[1], [2] Partly because of this, tobacco document research has significantly deepened understanding of the range of ways in which corporations use charitable donations to influence policy.[3], [4], [5], [6], [7], [8], [9], [10], [11], [12], [13], [14], [15], [16] This research suggests that tobacco companies primarily allocate charitable contributions on the basis of their potential to produce five proximate political effects: access to policymakers; constituency building amongst civil society organisations to build support for policy positions and generate third party advocacy;[17], [18], [19] constituency fragmentation[20] in which donations are used to weaken opposing constituencies; enhancement of the donor's status as a source of credible information, and framing/agenda setting (see Table 1).[13], [14], [15], [16]

**Table 1:** BAT Subsidiary Stakeholder and Social Reports Reviewed for Evidence of Political Philanthropy.

Country	World Bank Classification (gross national income per capita)[204], [205]	Report	Discussion of donations (detailed/moderate/minimal/none)	Framing/Agenda setting (Donations conveying a positive contribution to social and economic development (low/middle income countries) and economic regeneration (high income countries))	Framing/Agenda setting (Donations aimed at highlighting other risks to health)	Donations consistent with constituency building	Donations consistent with achieving access
Bangladesh	Low income	Social Report 2003–2005[145]	Moderate	Yes	No	Yes	No
East Africa	Low income	Social Report 2006/07[168]	Moderate	Yes	No	Yes	No
Uganda	Low income	Report to Society 2003[160]	Detailed	Yes	Yes	Yes	Yes
Nigeria	Lower middle income	Stakeholder Report 2010–2011[147]	Detailed	Yes	Yes	Yes	No
Pakistan	Lower middle income	Stakeholder Report 2008/09[159]	Detailed	Yes	Yes	Yes	No
Sri Lanka	Lower middle income	Social Report 2005/06[167]	Detailed	Yes	No	Yes	No
Russia	Upper middle income	Stakeholder Report 2009[206] and 2010/2011[149]	Minimal	Yes	No	No	No
Mexico	Upper middle income	Social Report 2006[169]	Moderate	No	No	Yes	No
South Africa	Upper middle income	Social Report 2008[111]	Detailed	Yes	Yes	Yes	No
Canada	High income	Social Report 2006/07[150]	Moderate	Yes	No	Yes	No
New Zealand	High income	Social Report 2007[148]	Detailed	No	No	Yes	Yes
Germany	High income	Stakeholder Report 2010[207]	None	No	No	No	No

This work has been instrumental in facilitating efforts to restrict the tobacco industry's ability to benefit politically from its charitable donations via Articles 5.3 and 13 of the World Health Organization's Framework Convention on Tobacco Control (FCTC), the World Health Organization's (WHO) first global public health treaty (Appendix S1). Nevertheless, existing studies have primarily focused on the US multinational Philip Morris (PM) and may provide an incomplete account of the political versatility of corporate philanthropy. The present study therefore aims to develop the existing literature in three respects. First, it aims to explore the extent to which existing research on tobacco industry philanthropy can be generalised by using industry documents to examine the political aims underlying British American Tobacco's (BAT) charitable contributions. Second, it aims to develop an integrated model of corporate political philanthropy that combines the results of the present documentary analysis with documentary findings of the existing literature. Third, it aims to examine the political aims behind contemporary examples of BAT charitable donations reported in the company's recent social and stakeholder dialogue reports and thereby evaluate the contemporary relevance of the model. In doing so, the paper aims to inform the implementation of Articles 5.3 and 13 of the FCTC by providing an in depth, synthesised analysis of the political aims underlying tobacco industry philanthropy.

## Methodology

The present study emerged from a larger programme of work that aimed to explore the rationale, extent and impact of BAT's Corporate Social Responsibility (CSR) activities. BAT is headquartered in the UK and is the third largest tobacco company by revenue, after Philip Morris International and the Imperial Tobacco Group.[21], [22] It represents itself as the most international tobacco company on the basis of its large number of subsidiaries in low and middle income countries and has a strong track record of providing money and gifts in kind to a wide variety of organisations.[23], [24], [25], [26]

Documents were identified via online searches of the Legacy Tobacco Documents Library (http://legacy.library.ucsf.edu) between June 2009 and May 2011. For the current study 459 documents were studied in detail and indexed using Endnote. Analysis of the documents was based on a qualitative, hermeneutic methodology [27] with documents coded according to an inductively developed framework drawing on concepts used to describe corporate political activity and policy influence in the social sciences such as access, direct lobbying, constituency building, third party lobbying, policy subsidies, and agenda setting.[20], [28], [29], [30], [31], [32], [33], [34], [35], [36], [37], [38], [39], [40] In addition, the documents were organised chronologically to draw out changes in the thinking underlying BAT's charitable contributions over time.

The political aims underlying BAT's contemporary record of charitable giving were examined using BAT's social, sustainability (hereafter referred to as social reports), and stakeholder dialogue reports. As of 12^th^ December 2012, BAT's Reporting Download Centre (http://www.bat.com/group/sites/uk__3mnfen.nsf/vwPagesWebLive/DO6RZGHL?opendocument&SKN=1) provided links to 12 group social reports, 6 group stakeholder dialogue reports, 7 subsidiary stakeholder reports, and 34 subsidiary social reports (published between 2002 and 2012). We downloaded 12 English language stakeholder dialogue and social reports produced by BAT subsidiaries covering the World Bank's four main country classifications (low income, lower middle income, upper middle income, high income). Selected reports were downloaded on 12^th^ December 2012 and searched using key terms such as charit∼, community, donat∼, social invest∼, and philan∼. Examples of charitable giving subsequently identified were then coded using the same concepts employed to categorise the documentary findings. The web-sites of charitable foundations referred to in the reports selected were also reviewed and coded using the same concepts.

## Results

### The Evolution of Strategic Philanthropy within BAT

BAT documents from the late 1970s and early 1980s suggest that although BAT internal guidelines on charitable giving aimed to tie charitable donations to the firm's broader commercial objectives,[41], [42], [43], [44] in practice, the broad construction of these policies meant that contributions were relatively unfocused[41], [42], [43] and donations were allocated to a wide range of causes and groups.[43], [45], [46], [47], [48], [49], [50], [51], [52] By the early 1990s concerns over increasing efforts by national governments to introduce public smoking restrictions combined with general disquiet over the anti-globalisation movement encouraged BAT staff to take a more methodical approach to managing the company's image and reputation[53], [54], [55], [56], [57], [58], [59], [60], [61] (we use reputation for both concepts to denote what third parties think of BAT).[53], [62], [63], [64], [65], [66] During the early 1990s the first evidence emerges of BAT staff emphasising the potential political value of philanthropy. Documents tie donations to the enhancement of the company's reputation, which was regarded as key to maintaining the company's political influence in existing markets and establishing it in countries which BAT was expanding into following trade reforms and privatisation of formerly state owned tobacco manufacturers.[54], [55], [56], [61], [63], [67], [68], [69], [70], [71], [72], [73], [74] More specifically, a positive reputation was linked to a number of discrete political objectives via a range of intermediate effects. These intermediate effects included increasing social actors' acceptance of company messages, placating social actors who might otherwise oppose the company, supporting constructive relationships with governments, and building support amongst local and national communities,[54], [55], [56], [58], [60], [61], [68], [69], [71], [75], [76], [77], [78], [79], [80], [81] whilst objectives encompassed changes in excise systems, the successful defence of product liability litigation, general regulatory management, and the facilitation of joint ventures and market entry.[58], [59], [70], [79], [82], [83], [84], [85]

A review conducted in the late 1990s by consultant Julian Oliver, who had worked with Shell on its CSR programme, suggests that BAT was slow to harness the political potential of charitable contributions.[86] Oliver concluded that the company's contributions were largely reactive and disjointed and, therefore, generated “little brand or corporate reputational returns”.[45], [46], [47], [48], [49], [87], [88] He advocated a more “integrated” and “strategic” approach organised around a small number of common themes.[87] By tailoring these themes to local demands, Oliver argued that BAT could convey a “common message to public policy makers, international NGOs and the media” that the company understood its communities and customers better than other social actors.[87] The key, Oliver claimed, was to identify and support projects that had “a high political priority” which would “enhance BAT's ability to build ‘platforms for dialogue’ with rule-makers” and “deliver tangible benefits in terms of improved access, influence and international recognition/reputation”.[87]

The trigger for Oliver's report seems to have been concern over the company's declining political influence.[89], [90], [91], [92], [93], [94], [95] BAT attributed this to failings in its existing political strategy, which it regarded as reactive and confrontational.[90], [95] Its managers, therefore, advocated a different approach that centred on repackaging its “philanthropic activities” to actively change negative perceptions of the company.[95] The evidence suggests BAT accepted Oliver's analysis of the political underperformance of the firm's philanthropy and introduced concrete changes reflecting its managers' faith in the politically restorative powers of strategic giving.[94], [96], [97], [98] At around the same time Oliver presented his report to the company, BAT began to make large, conspicuous donations to education institutions, health organisations and NGOs.[16], [99], [100], [101], [102], [103], [104], [105], [106], [107] More recent evidence suggests that large donations around specific themes continue to characterise BAT's strategy for allocating charitable contributions (see, for example, Appendix S2).[108], [109], [110], [111], [112]

### Political Aims of BAT's Charitable Donations

#### Constituency Building

The capacity of donations to “develop potential allies” has been a major theme within BAT's charitable giving with contributions being made to build constituencies with a wide range of groups, including: communities local to company plants, constituencies that make up parts of the tobacco supply chain (such as leaf growing communities), and civil society organisations (see Appendices S2 and S3).[98], [113] Donations have been considered key to building constituencies partly by facilitating closer relationships and, thereby, greater trust with recipients[113] and partly by encouraging recipient organisations, their beneficiaries and other constituencies that benefit indirectly from contributions (such as local communities) to identify their interests with those of BAT.[90], [113]

Documents suggest that building constituencies to expand opportunities for third party lobbying has been closely linked to concerns about the company's declining credibility as a source of policy relevant information.[86], [113] Thus, BAT supported the Beijing Liver Foundation (renamed the Beijing Health Promotion Society in 1999 – see below) to lobby the Ministry of Public Health to “maintain a perspective on health issues”, recognising that the company could not “credibly, directly communicate with the Ministry.”[114], [115] Donations capable of sustaining partnerships have been particularly valued within BAT for their capacity to facilitate third party lobbying, reflecting a conviction in the effectiveness of long term relationships to foster trust both with partnering organisations and other NGOs and public officials within the partners' networks.[81], [113]

#### Access and Relationship Building

BAT documents highlight the perceived value of philanthropy in securing access to public and elected officials by generating political capital and goodwill amongst policy élites, creating opportunities to meet with them, and fostering trust amongst other social actors, such as NGOs and opinion formers (see Appendices 2 and 3).[58], [80], [113], [116] In addition, there are examples of BAT specifically allocating money to form partnerships with NGOs in the hope of exploiting their links with policymakers and contributing to programmes with a view to entering into direct partnership with government ministries(Appendix S2).[117]

There is also evidence of an intention to earmark donations for specific causes, which overlap with government policies, precisely because of their potential to facilitate access to policymakers.[86], [113] One document reported that the “essence” of targeting charitable contributions was to “identify and support projects that have high political priority and that would enhance BAT's ability to build ‘platforms for dialogue’ with rule-makers in several countries.”[87], [98] For example, BAT's decision to fund urban regeneration projects and City Technology Colleges in the UK in the late 1980s and early 1990s, was rationalised on their “proximity and access to the UK government”.[94]

Financial support for scholarships aimed at creating supportive political constituencies demonstrates the long term and sophisticated nature of the use of philanthropy to optimise access. One report from the late 1990s, for example, highlighted the importance of providing finance for overseas postgraduate students as part of a “long-term investment in potential leaders in developing markets”,[94] whilst another (dated 1999) explained that funding scholarships in tertiary education would create “alumni that will in future be part of the national leadership of the world in which we do business.”[118]

#### Framing Effects and Agenda Setting

Documents highlight several ways in which donations are used to shape how the company is perceived (reputational framing effects). For example, an early communication plan for the Tobacco Advisory Council (TAC) (of which BAT was a member) refers to using “philanthropic work as a means of demonstrating industry concern for social well-being, and of offsetting negative effects accruing to the industry from the primary health and passive smoking issues”.[119] Existing research suggests that mitigating negative assessments of the firm has the potential to shape the tobacco control agenda by changing how policymakers and civil society organisations assess its aggregate social impacts. [5], [13], [30], [120], [121], [122], [123], [124], [125], [126]

BAT's documents also indicate that the company has consistently linked contributions to the “needs and aspirations of national communities which are relevant to local development needs”.[59], [68], [69], [94], [113], [127], [128] Existing studies on the political effects of displacement frames (which work to change perceptions by providing alternative ways of appraising issues, rather than directly challenging the facts that underlie them) suggest that donations which consistently produce this association have the potential to shape the tobacco control agenda by shifting the primary basis upon which the firm should be judged: away from health towards its perceived economic impacts on inward investment, employment, and foreign earnings.[5], [13], [30], [120], [121], [122], [123], [124], [125], [126], [129]

The documents indicate that BAT has tried to produce a similar effect by associating the firm with NGOs involved in development (see Appendix S2).[130] A 2000 presentation by Andreas Vecchiet, then BAT's International Political Affairs Manager, highlighted the importance of partnerships to promoting “the proposition that the answers to major concerns arising from perceived market and governmental failure can be reached via bona fide and mutually beneficial partnerships between governments, companies and the ‘civil society’”.[131] The slides explain that the “subtext” to such partnerships was that “profits from legal products…are a precursor of and underpin political, social, economic and environmental development”.[131]

In addition, the documents indicate a close connection between constituency building and agenda setting with donations allocated to some groups on the basis of their potential to shape policy agenda though their influence on government thinking and news reporting.[16], [99], [131], [132], [133], [134], [135], [136], [137] Furthermore, donations have been made to shift thinking on the importance of tobacco control regulation by influencing perceptions of the relative risks of tobacco to population level health. The approach was originally part of BAT's efforts to limit the spread of smoking restrictions and involved the company highlighting specific (non-tobacco related) health concerns to focus attention on “real community health concerns.”[138], [139] BAT China's financial support for the Beijing Liver Foundation indicates how the strategy has informed decisions on charitable giving. Founded in 1997 by the Soong Ching Ling Foundation (now the China Soon Ling Foundation),[140], [141] a high profile and well-connected Chinese charity which BAT considered to be a key anti-smoking group in China,[128] BAT's support for the Beijing Liver Foundation was designed to raise the profile of hepatitis which it considered “should be of greater significance to the [People's Republic of China] and the WHO” than smoking.[142] According to one document, the ultimate aim of the donation was to “reprioritise the agenda” of both the Soong Ching Ling Foundation and the Ministry of Public Health and “divert the public attention from smoking and health issues to liver diseases”.[115], [140], [143]

Finally, documents also suggest donations are used to neutralise the agenda setting potential of civil society organisations calling for regulation to address the socially harmful consequences of corporate activity. BAT's donation to the Earthwatch Institute (Europe) in the early 1990s, for example, seems to have been partly motivated by a desire to defuse calls for more rigorous, formal, regulation of corporate environmental harm in the lead up to the 1992 United Nations Conference on Environment and Development (the Rio Summit) (Appendix S2).

#### Subsidies and Direct Political Leverage

A 1994 letter from Raymond Acorda, Managing Director of the Bangladesh Tobacco Company, to the chair of Bangladesh's National Board of Revenue illustrates that BAT has used contributions in low income countries as a bargaining chip in its efforts to change policy. In the letter Acorda indicated that a failure to reverse an increase in the tobacco excise rate might lead to a withdrawal of charitable donations and sponsorships.

“If these two brands continue to be adversely affected we will be unable to continue to cover our Production, Administrative and Selling overheads - furthermore the cash will not be available in order to invest in the cigarette business; in some of the diversification projects (e.g. Sunflower oil) which we have been experimenting with; and much of the social and voluntary work that BTC [Bangladesh Tobacco Company] supports (e.g. afforestation, charitable donations, sponsorships etc.).” [144]

This illustrates the potential for charitable donations to be used as financial subsidies that create direct political leverage. A discussion amongst BAT managers over how to replicate the perceived political benefits of a donation by R.J. Reynolds to repair the Haizhu Bridge in Guangzhou province, China, in the early 1990s, suggests one way in which donations might produce this effect is by creating a sense of indebtedness amongst political élites.[143] BAT managers noted that the donation constituted the “sort of gesture to which officialdom will feel obligated”.[143]

#### Enhancement of Company Status as a Source of Credible Information

BAT documents indirectly link charitable donations to enhancing the company's status as a source of credible information through their positive effect on corporate reputation.[58], [78] Our documentary searches failed to locate specific examples of donations being made to produce this effect, and the potential for philanthropy to increase the company's credibility as a purveyor of information may, therefore, have represented a general underlying justification of and guide for donations within the company.

### Evidence of Political Philanthropy in BAT's Contemporary Charitable Giving

The review of selected social and stakeholder dialogue reports indicates that donations are still being used to produce a similar range of political effects (see Table 1). In reports produced by subsidiaries in low and middle income countries, philanthropy is used extensively to link the company to social and economic development (see Table 1). In some reports the association is implied by the nature of the donation and context in which it is made. This is illustrated by BAT Bangladesh's coverage of its provision of information technology education to the rural poor. The account emphasises the world class environment and standard of the education, which actively seeks to help young people to accomplish things “they never dreamt of”, and highlights the large number of students who have obtained work as a result.[145] Likewise, the Pakistan Tobacco Company highlights the value of its mobile doctors in providing free health care “where there are little or no medical facilities”[146] and the role of its free Learning Resource Centres which “contribute towards the development of skilling resource in the country.”[146]

Other company reports are more explicit about the role of their donations in facilitating development. BAT's South African Sustainability Report for 2008, for example, notes that its contributions are designed to “improve the economic, social and environmental sustainability of previously disadvantaged individuals and communities” (see also below).[111] BAT Nigeria, which channels its philanthropy through the BATN Foundation, extends this theme by illustrating how different aspects of its philanthropy support the Nigerian Government's drive to achieve the Millennium Development Goals, such as the eradication of extreme poverty and hunger and environmental sustainability.[147] The subtext of this narrative - the rejection of the idea that the tobacco industry encumbers social and economic development - is made explicit in the company's claim that it is leading “a collective private sector approach to sustainable social development” which will minimise poverty and empower Nigerians to “own and control their economic destiny.”[147] In high income countries, donations to social and economic projects are commonly aimed at ameliorating the social dislocation caused by deindustrialisation (focusing, for example, on training, economic regeneration, and the alleviation of poverty and social exclusion).[148], [149], [150]

There is also evidence of BAT using donations to highlight non tobacco risks to public health in line with previous attempts to reorder policymakers' sense of public health priorities. The evidence is particularly strong in the case of South Africa where BAT focuses its donations on alleviating HIV/Aids in the country's disadvantaged communities through the BAT South Africa Signature Trust.[151], [152], [153], [154] In addition, the company's Significant Endemic Disease Programme which aims to reduce the impact of malaria, tuberculosis, AIDS, hepatitis and significant bowel infection amongst the company's employees, their families and communities operated in 21 countries (Bangladesh, Cambodia, China, Eritrea, Greece, Indonesia, Kenya, Malaysia, Mexico, Mozambique, Nigeria, Pakistan, Russia, Samoa, South Africa, Taiwan, Uganda, UK, Ukraine, Vietnam and Zimbabwe, with a major focus on HIV/AIDS in Africa, the Middle East and Asia-Pacific) in 2010.[155], [156], [157], [158]

BAT subsidiaries' widespread practice of making contributions to improve water quality (through, for example, water filtration plants in Pakistan and drilling bore holes in Uganda and Nigeria) may have the same effect.[147], [159], [160], [161], [162] Pakistan Tobacco Company's investment in water filtration plants, for example, is part of a broader programme aimed at increasing awareness of the benefits of clean drinking water and takes effect against a backdrop of high levels of mortality resulting from poor quality drinking water.[163], [164], [165], [166] In the absence of contemporary industry documentary evidence, the motivation behind these initiatives is difficult to discern. At the very least, however, they are likely to send mixed messages about the role of the tobacco industry in mortality in low and middle income countries.

BATN Foundation's practice of aligning charitable donations with the Nigerian government's objectives of achieving the Millennium Development Goals by 2015 echo donations made in the 1990s aimed at synchronising donations with government priorities in order to secure access to policy élites.[147] Similarly, donations made available to projects in areas local to manufacturing plants and to tobacco farmers are consistent with donations made in the 1990s aimed specifically at building constituencies. These are common and have included medical assistance (Sri Lanka) and community water projects in regions associated with tobacco farming (Sri Lanka and East Africa) and support for social and recreational projects in areas close to manufacturing plants and area offices (Canada, Mexico, and New Zealand).[148], [150], [167], [168], [169]

Although using donations aimed at building constituencies can indirectly weaken opposing political constituencies by limiting the pool of willing allies, there were no clear examples of donations being used to reduce the strength of opposing political constituencies (constituency fragmentation) or generate direct political leverage. Evidence of this is rarely publicly available and is unlikely to be published in social and stakeholder dialogue reports. This is consistent with BAT guidance on charitable donations from the 1990s which emphasised the importance of ensuring that the political drivers of donations did not become public knowledge.[71]

## Discussion and Conclusions

The study identifies several ways in which philanthropy is considered to work politically either as a technique in its own right (as in the case of sponsored events which provide opportunities for access with policy élites) or by facilitating more traditional political tactics (as happens, for example, where charitable donations are considered to make access to policy élites more likely by creating goodwill).[5], [6], [7], [13] The documentary analysis identified four aims (constituency building, access and relationship building, enhancement of the company's status as a source of credible information, and framing effects/agenda setting) already outlined in the existing literature,[13], [14], [15], [16] and one new aim (subsidies/direct political leverage) (Table 2). Our searches did not, however, identify constituency fragmentation (see Table 2), which McDaniel and Malone[15] found as a political aim underlying aspects of PM's philanthropy, although this may be an incidental effect of BAT's work around child labour[170] and with local communities (see, for example, Appendix S3 and the section above). Donations in these contexts indirectly weaken opposing political constituencies by creating disincentives to collaborate with public health advocates amongst recipient organisations.

**Table 2:** Combined Political Aims underlying Tobacco Industry Philanthropy identified in the Present Study and Existing Studies.

Underlying Aims	Explanation of Effect	Existing Literature	Present Study
Constituency Building	Donations used to facilitate closer relationships with recipient organisations by generating trust and support and shape their organisational priorities. Organisations are encouraged to lobby and advocate on behalf of the industry, thereby expanding political conflicts around tobacco control.	Tesler and Malone, 2008; McDaniel and Malone, 2009 and 2011	Yes
Constituency Fragmentation[20]	Donations used to dissuade recipient organisations from lobbying against companies' interests. It has broadly the opposite effect to constituency building in that it is designed to contain political conflicts by weakening constituencies opposed to the tobacco industry.	McDaniel and Malone, 2009	
Access and Relationship building	Donations used to facilitate access both directly (by creating opportunities to meet with policymakers by: securing invitations to charity events patronised by officials and their spouses; inviting them to corporate sponsored charitable events; targeting charities which overlap with government priorities; and creating partnerships with politically connected recipient organisations) and indirectly (by generating political capital and goodwill amongst policymakers; strengthening relationships with policy élites; and fostering trust amongst NGOs and opinion formers).	Tesler and Malone, 2008; McDaniel and Malone, 2011.	Yes
Subsidies and Direct Political Leverage	Political leverage achieved by creating a sense of indebtedness through the provision of financial subsidies to specific political projects.		Yes
Enhancement of the Company's status as a source of credible information	Credibility as a source of information, data or evidence is linked to positive corporate reputation. This effect is designed to revive, maintain and, potentially in some cases, enhance the company's underlying structural information advantage in policymaking.	Tesler and Malone, 2008	Yes
Agenda and framing effects	Donations to specific causes or aimed at building partnerships with specific NGOs associate the company with economic and social development with a view to shifting thinking on the policy importance of regulating the market environment for tobacco.		Yes
	Donations to charities involved in combatting non tobacco related risks to health are made to reprioritise perceptions of the relative risks of smoking on population level health.	Muggli, *et al*, 2008	Yes
	Donations are channelled towards some NGOs in order to neutralise the agenda setting potential of civil society organisations.		Yes
	Donations are used to shape how the company is perceived (reputational framing effects) which mitigate negative assessments of the firm and change how policymakers and NGOs assess the aggregate social impacts of the firm.		Yes

Charitable donations recorded in recent BAT social reports bear close similarities with these political aims. In particular, the reports provide a rich store of contemporary examples of donations that may help shape the tobacco control agenda. In practice, the political aims of donations overlap and are likely to be mutually reinforcing. Efforts to build constituencies, for example, can produce both agenda setting and framing effects, whilst changes in how social actors think about the industry and its products (framing effects) are likely to facilitate other more immediate political aims, such as access and constituency building. Further, in the case of scholarships, donations seem to be partly allocated on the basis of their capacity to build constituencies which may facilitate future access to policy élites. By highlighting these interconnections, the similarities between BAT and PM's political use of philanthropy, and the likely political aims underlying contemporary BAT donations, the paper provides strong evidence of the need for Parties to the FCTC to ban tobacco industry philanthropy outright.

### Contemporary Value of Political Philanthropy to Tobacco Companies

Philanthropy represents a particularly valuable political tool for contemporary transnational tobacco companies for three reasons. The first concerns the on-going deterioration in the industry's insider status and decline in its reputation as a reliable provider of policy relevant information.[95], [171], [172] Philanthropy has the potential to offset the former and reverse the latter by creating a less contentious basis for access which, over time, can facilitate more routine interactions. This creates trust and strengthens relationships between companies and officials, which is likely to enhance firms' status as credible purveyors of information and augment their information advantage in policymaking.[13], [173], [174], [175], [176]

The second concerns the role of charitable donations in building constituencies, an effective means of political influence that strengthens the effectiveness of other business political activities and expands political conflicts in which business has an interest.[17], [18], [19], [28], [177] Building constituencies through philanthropy can potentially circumvent the reluctance that many economic and civil society actors are likely to have in openly collaborating with the tobacco industry[113] and, by stimulating third party advocacy and indirect lobbying, provides a potential means of avoiding transparency rules recommended by the Guidelines for Implementation of Article 5.3 of the FCTC.[178]

Finally, philanthropy has the potential to neutralise on-going work aimed at highlighting the net negative social and economic impacts of the tobacco industry, which has been a key driver of efforts to regulate the industry under the FCTC.[128], [179], [180], [181], [182] Charitable donations are a form of symbolic communication which have the potential to change perceptions through the associations they create. By representing tobacco companies as important vehicles of sustainable development, philanthropy has the potential to stymie continuing efforts to model the net negative economic effects of the tobacco industry in low and middle income countries and reinforces manufacturers' historic efforts to create different ways of thinking about the industry and the risks it poses to the broader public welfare.[183] Research in other policy domains suggests that this may be particularly effective at facilitating BAT's other political activities as it asks social actors to focus on a different set of questions about the industry, rather than directly challenging their existing beliefs about its role in propagating tobacco related disease.[129]In higher income countries the focus of BAT's donations on training, economic regeneration, poverty and social exclusion support the company's efforts to shape the tobacco control agenda in a broadly similar way. By emphasising that it provides capital for programmes which ameliorate the social effects of deindustrialisation, these types of donation convey the continuing relevance of the company to the long term social and economic success of richer nations.

### Strengths, Limitations and Further Research

Despite the considerable epistemological advantages that tobacco industry documents offer in analysing corporate decision-makers' thinking on charitable contributions they rarely depict the outcome of tobacco companies' political activities. Whilst further research may improve our understanding of the political effects of tobacco industry philanthropy, these are likely to be difficult to analyse given that they take place under low levels of visibility and are likely to take effect over long periods of time. This was recognised by BAT managers in the present study who noted that actions undertaken to improve a firm's image and reputation were only likely to show results after several years of “continuous and consistent efforts of many years.”[184] A further methodological complication concerns the context dependent nature of these effects. The ability of donations to influence social actors' perception of a company are generally considered to be more potent where stakeholders are not locked into a particular set of beliefs about a company or where other measures of a company's social utility and underlying values are unclear, underdeveloped or contradictory.[5] This may explain the importance BAT has attached to using philanthropy in shaping perceptions in new markets[56] and it is consistent with previous research indicating the importance BAT managers have attached to using CSR practices to counter the spread of critical perceptions of the industry from high to low and middle income countries.[95]

There are also a number of weaknesses in using social and stakeholder reports as a basis for coding charitable gifts. First, donations are usually only covered in stakeholder reports where they are relevant to issues raised in dialogue with stakeholders. Second, contributions are not necessarily discussed in sufficient detail for coding. Third, the underlying motivation of charitable donations or their political effects (such as whether donations have created occasions for BAT managers to meet with policymakers) is not discussed in the reports. News reports of donations being used as financial inducements to political élites aimed at steering them towards particular decisions suggests that in depth investigations relying on a mix of methodologies (such as semi-structured interviews, forensic accounting, and documentary work) may address these weaknesses.

### Policy implications

By confirming the findings of earlier work on PM,[13], [14], [15], [16] the present study underlines the importance of full implementation of Article 13 of the FCTC which provides a unique opportunity to outlaw socially suboptimal philanthropy (Appendix S1). Unlike Article 5.3, Article 13 has the advantage of preventing political impacts that occur even when news of gifts are not widely publicised as in the case of donations to politicians' favoured charities. Civil society organisations' dependence on industry philanthropy potentially represent an important obstacle to its prohibition. In Russia, for example, charities' responses to reports of a possible ban on tobacco industry donations were highly critical, reflecting concerns that revenue streams to unfashionable charities, such as those involved in caring for the elderly, might be affected.[185], [186] These criticisms are likely to be particularly resonant in low and middle income countries where state social provision is less developed. Such criticism underlines the importance of clear communication and alliance building between health professionals and civil society organisations, as well as the strategic value of compensating affected charities, which could be achieved through a hypothecated tax on tobacco companies.[187]

In addition to advocating legal reform, awareness raising of the motivations underlying industry philanthropy is paramount. Officials' and the public's estimation of the motives underlying charitable donations are considered central to their capacity to shape impressions.[5], [11], [13] Consequently, challenging this frame is likely to reduce their political impact. The model developed in the present study can facilitate this process by providing a framework for identifying the potential political objectives of donations that are not immediately apparent, such as agenda setting and constituency building.

A final question concerns whether this model can be applied to companies in other industrial sectors which have negative impacts on health, such as food and alcohol.[188], [189], [190], [191], [192], [193] Corporations' political strategies are context dependent and vary according to a wide range of institutional factors including levels of regulatory risk and political trust.[9], [11], [28], [194], [195], [196], [197], [198], [199] Political strategies used by tobacco companies are, therefore, likely to be different from those operating in other sectors of the economy. However, a recent donation of US$10 million by the American Beverage Association (the trade association of the soft drinks industry) to the Children's Hospital of Philadelphia confirms the findings of other research underlining the political value of charitable donations and CSR across industrial sectors.[4], [200], [201] The donation followed testimony to the City Council by doctors from the Children's Hospital of Philadelphia about the dangers of sugar-sweetened drinks. At the time the City Council was considering whether to introduce a tax on sugar-sweetened beverages sold in the city,[202] suggesting very strongly that the donation was designed to fragment and, therefore, weaken the political constituency in favour of policy change. This and other examples[203] strongly suggest that the model developed here can be used by public health professionals to interpret the political motivations (and potential effects) of charitable giving in the food, soft drinks, and alcohol sectors.

## Supporting Information

Appendix S1 Articles 5.3 and 13 of the Framework Convention on Tobacco Control.
(DOCX)

Appendix S2 The BAT Biodiversity Partnership.
(DOCX)

Appendix S3 The Pécs Diagnostic Centre and other Charitable Projects funded by BAT Pécsi Dohánygyár (BAT's Hungarian subsidiary).
(DOCX)
