# Supplementary material for: Corporate Philanthropy, Political Influence, and Health Policy
Source: PLoS One. 2013 Nov 27;8(11):e80864. doi: 10.1371/journal.pone.0080864 (PMC3842338; doi:10.1371/journal.pone.0080864)
Supplement: Appendix S1 — Articles 5.3 and 13 of the Framework Convention on Tobacco Control. (DOCX) [file pone.0080864.s001.docx]

**Appendix S1. Articles 5.3 and 13 of the Framework Convention on Tobacco Control**

Article 5.3 of the FCTC specifically requires parties to protect public health policy from commercial and other vested interests of the tobacco industry.[[1](#_ENREF_1)] Guidelines for implementation of Article 5.3 recommend a number of activities aimed at regulating activities described as “socially responsible” by the tobacco industry. The most relevant of these to industry philanthropy are recommendations 6.3 and 6.4. Recommendation 6.3 advises parties to prevent the tobacco industry from disclosing either activities described as socially responsible or the expenditures made for these activities. 6.4 urges parties to prevent acceptance by any branch of government or the public sector of political, social, financial, educational, community or other contributions from the tobacco industry or from those working to further its interests.

Article 5.3 does not recommend that parties to the Convention ban industry charitable donations outright and, therefore, does not preclude tobacco companies from using donations politically. However, Article 13 requires parties to ban all forms of sponsorship which is defined in Article 1(g) to include financial and in-kind contributions to community, health, welfare and environmental organisations.[[2](#_ENREF_2)] Progress in implementing this provision has been slow.[[3](#_ENREF_3),[4](#_ENREF_4),[5](#_ENREF_5),[6](#_ENREF_6),[7](#_ENREF_7)] with only Mauritius having introduced legislation capable of formally outlawing charitable donations.[[8](#_ENREF_8)]

1. Smith KE, Gilmore AB, Fooks G, Collin J, Weishaar H (2009) Tobacco industry attempts to undermine Article 5.3 and the “good governance” trap. Tobacco Control 18: 509-511.

2. World Health Organization (2008) Guidelines for implementation of Article 13 of the WHO Framework Convention on Tobacco Control (Tobacco advertising, promotion and sponsorship).

3. Corporate Accountability International (2012) Big Tobacco CSR Violates Ad Ban.

4. Corporate Accountability International (2012) Cutting through the Smoke.

5. Corporate Accountability International (2010) Corporate Accountability International and NATT Commend the Phillipines for Protecting Against Tobacco Industry Interference.

6. African Tobacco Control Consortium (2012) Tobacco Watch: Africa - Monitoring Countries' Performances on the Global Tobacco Control Treaty.

7. Southeast Asia Tobacco Control Alliance (2012) End Tobacco Industry Corporate Giving. Thailand: SEATCA.

8. Minister of Public Health (2008) Regulations made by the Minister under sections 193 and 194 of the Public Health Act.
