# Supplementary material for: Corporate Philanthropy, Political Influence, and Health Policy
Source: PLoS One. 2013 Nov 27;8(11):e80864. doi: 10.1371/journal.pone.0080864 (PMC3842338; doi:10.1371/journal.pone.0080864)
Supplement: Appendix S2 — The BAT Biodiversity Partnership. (DOCX) [file pone.0080864.s002.docx]

**Appendix S2. The BAT Biodiversity Partnership**

In 1990, BAT became a regular donor of the Earthwatch Institute (Europe) (EIE), a medium sized environmental charity involved in the preservation of biodiversity with a strong track record of co-operating with business.[[1](#_ENREF_1)] Company documents suggest that donations were likely to have been partly made in the hope of neutralising NGO efforts to pressure the company into more far-reaching environmental commitments.[[2](#_ENREF_2)]

In the late 1990s, BAT entered into the BAT-Earthwatch Partnership with EIE (now the BAT Biodiversity Partnership) and a number of other NGOs which involved a much larger donation [[3](#_ENREF_3),[4](#_ENREF_4)]. Documents suggest the donation was made in the expectation of creating a global ally in developing countries,[[5](#_ENREF_5),[6](#_ENREF_6)] facilitating access to government officials, and building relationships with other NGOs.[[5](#_ENREF_5),[6](#_ENREF_6),[7](#_ENREF_7),[8](#_ENREF_8)] Both the British Government and European Commission contributed to EIE’s African Fellowship Programme and a BAT memo identified the Fellowship Programme as a potential opportunity to gain access to the UK government’s department for international department.[[9](#_ENREF_9)] BAT had identified NGOs as an important source of influence over the public, media coverage and public and elected officials[[6](#_ENREF_6),[10](#_ENREF_10),[11](#_ENREF_11),[12](#_ENREF_12),[13](#_ENREF_13),[14](#_ENREF_14)] and was concerned about their increasing success in shaping policy on issues concerning multinational corporations.[[6](#_ENREF_6),[15](#_ENREF_15)] Company managers hoped that fostering greater trust with NGOs would improve BAT’s access to government officials and multilateral organisations and ultimately help it to promote a model of development which emphasised the importance of “profits from legal products” as a precondition of “political, social, economic, and environmental development”.[[6](#_ENREF_6),[7](#_ENREF_7),[8](#_ENREF_8),[14](#_ENREF_14),[15](#_ENREF_15),[16](#_ENREF_16),[17](#_ENREF_17)]

Engagement with NGOs was also part of BAT’s strategy to secure consent for its self-regulatory alternative to the FCTC.[[18](#_ENREF_18)] It hoped to communicate its relationships with NGOs to “show that BAT [was] acting responsibly on social and environmental agendas”, promote the ideas that tobacco companies could be self-regulating[[7](#_ENREF_7),[9](#_ENREF_9),[18](#_ENREF_18),[19](#_ENREF_19)], and that civil society should not impose European standards of behaviour on developing countries. In short, partnerships with NGOs aimed to “demonstrate the proposition that the answers to major concerns arising from perceived market and governmental failure can be reached via….mutually beneficial partnerships between governments, companies and the `civil society"'.[[8](#_ENREF_8),[19](#_ENREF_19),[20](#_ENREF_20)]

1. (1998) Corporate Environmental Responsibility Group: Report on Activities: 1997. British American Tobacco. pp. 321306277-321306283.

2. (1991) Environmental review; September 1991. British American Tobacco. pp. 201814439-201814469.

3. Oliver J (1998) Draft Corporate Responsibility Budget. British American Tobacco. pp. 322121456-322121460.

4. Oliver J (1998) Corporate Responsibilities Programme. British American Tobacco. pp. 322121448-322121455.

5. Opukah S (1999) Meeting with Earthwatch - Briefing Paper. British American Tobacco. pp. 321306472-321306478.

6. McDaniel PA, Malone RE (2011) British American Tobacco's partnership with Earthwatch Europe and its implications for public health. Global Public Health 7: 14-28.

7. (2000) The CORA Roadmap: CORA Strategic Steering Group. British American Tobacco. pp. 325136086-325136276.

8. Vecchiet A (2000) NGO Engagement. British American Tobacco. pp. 760066372-760066395.

9. Opukah S (1999) Earthwatch. British American Tobacco. pp. 321306480-321306482.

10. Vecchiet A [Notes from Andreas Vecchiet regarding role of multilateral UN agencies]. British American Tobacco. pp. 325113598-325113600.

11. (2000) CORA 2000 Project. British American Tobacco. pp. 325105410-325105423.

12. (1997) NGOs in Asia and Australia: How to Understand and Deal with them by Andreas Vecchiet. British American Tobacco. pp. 325134814-325134821.

13. British-American Tobacco: NGO Mapping Project. British American Tobacco. pp. 325134796-325134799.

14. Gonzalez M, Green LW, Glantz SA (2012) Through tobacco industry eyes: civil society and the FCTC process from Philip Morris and British American Tobacco's perspectives. Tobacco Control 21: e1.

15. Honour H Multinational and Social Accountability. British American Tobacco. pp. 322121600-322121608.

16. (1998) Corporate and Regulatory Affairs: Company Plan 1999 - 2001. British American Tobacco. pp. 321324291-321324477.

17. CORA Roadmap 2000-2001 Summary. British American Tobacco. pp. 325136079.

18. Marshall A (1999) The WHO Tobacco Free Initiative Establishing a Regulatory Body. British American Tobacco. pp. 321711653-321711667.

19. Weishaar H, Collin J, Smith K, Gruning T, Mandal S, et al. (2012) Global health governance and the commercial sector: a documentary analysis of tobacco company strategies to influence the WHO framework convention on tobacco control. PLoS Med 9: 26.

20. (2000) Martin Broughton Warns against 'New Colonialism' "Responsibility Provides our License to Operate". British American Tobacco. pp. 325242958-325242982.
