# Supplementary material for: Corporate Philanthropy, Political Influence, and Health Policy
Source: PLoS One. 2013 Nov 27;8(11):e80864. doi: 10.1371/journal.pone.0080864 (PMC3842338; doi:10.1371/journal.pone.0080864)
Supplement: Appendix S3 — The Pécs Diagnostic Centre and other Charitable Projects funded by BAT Pécsi Dohánygyár (BAT's Hungarian subsidiary). (DOCX) [file pone.0080864.s003.docx]

**Appendix S3 The Pécs Diagnostic Centre and other Charitable Projects funded by BAT Pécsi Dohánygyár (BAT’s Hungarian subsidiary)**

The Pécs Diagnostic Centre or Clinic was established in 1994 with funding from BAT Pécsi Dohánygyár, BAT’s Hungarian subsidiary. Located at Pécs Medical University the centre included diagnostic equipment (for cardiovascular disease and cancers) which was in short supply in Hungary. This enabled BAT to describe the Clinic as a unique, forward-looking public-private initiative which represented a major investment in the social and economic development of the country.[[1](#_ENREF_1),[2](#_ENREF_2),[3](#_ENREF_3),[4](#_ENREF_4),[5](#_ENREF_5)] The investment earned BAT’s Managing Director in Hungary an award for contributing to the social and cultural life of Pécs.[[5](#_ENREF_5)]As the largest private Clinic in Hungary, it was hoped that the Centre would give BAT Pécsi Dohánygyár “continuous positive publicity and press coverage.”[[6](#_ENREF_6)] Along with contributions to other causes (such as accommodation for the homeless, the arts and educational projects) the clinic was considered to “win allies in local markets…[and]…but open the doors of politicians and regulators.”[[5](#_ENREF_5)]

1. (1995) BAT Bulletin - February 1995. British American Tobacco. pp. 400849455-400849477.

2. Jennings M (0000) Successful Two Years. British American Tobacco. pp. 304024215-304024218.

3. (0000) BAT Industries: Community News. British American Tobacco. pp. 601041585.

4. Rudge L, Rudge L (1994) [Note regarding Scientific Research Group Meeting]. British American Tobacco. pp. 500833961-500833963.

5. Honour H (1998) Future of the Donations Budget. British American Tobacco. pp. 322121552-322121573.

6. (0000) The Pecs Diagnostic Clinic. British American Tobacco. pp. 500818091-500818097.
